# Eribulin and sintilimab combined with radiotherapy in a heavily pretreated patient with advanced retroperitoneal myxoid pleomorphic liposarcoma: a case report and literature review

**DOI:** 10.3389/fonc.2025.1719603

**Published:** 2025-12-05

**Authors:** Hongyu Zhuo, Dongna Li, Yang Fu, Xinyao Zhang, Xin He, Ying Xu, Jie Liu, Yu Jiang, Yaotiao Deng

**Affiliations:** 1 Department of Medical Oncology, Cancer Center, West China Hospital, Sichuan University, Chengdu, China; 2 West China Clinical Medical College, Sichuan University, Chengdu, China; 3 Department of pathology, West China Hospital, Sichuan University, Chengdu, China

**Keywords:** myxoid pleomorphic liposarcoma, eribulin, PD-1 inhibitor, pathology, partial response

## Abstract

Myxoid pleomorphic liposarcoma (MPLPS) is an ultra-rare subtype of soft tissue sarcoma. The limited number of reported cases has led to significant challenges in its clinical management. Here, we present the case of a 52-year-old woman diagnosed with advanced retroperitoneal MPLPS. As first-line therapy, she underwent 4 cycles of chemotherapy with doxorubicin and ifosfamide. Anlotinib was subsequently added to the first-line regimen. Following this, she received gemcitabine plus albumin paclitaxel as second-line therapy. Notably, a partial response (PR) was achieved and sustained for 12 months following the administration of eribulin and sintilimab combined with radiotherapy. The demonstrated effectiveness of this multidisciplinary approach underscores its potential as a novel treatment strategy for patients with advanced MPLPS. To our knowledge, this article represents the first detailed documentation of multidisciplinary diagnosis and treatment of MPLPS.

## Introduction

Myxoid pleomorphic liposarcoma (MPLPS) was officially classified as a distinct subtype of liposarcoma in 2020 World Health Organization (WHO) classification, characterized by unique morphological, immunohistochemical and ultrastructural features (1). Literature review reveals a limited number of case reports focusing on MPLPS (2–4). Advanced MPLPS presents a particularly unfavorable prognosis compared with other subtypes of liposarcoma, and there is currently no standardized treatment protocol for this disease (5).

In the landscape of liposarcoma treatment, eribulin, a structurally modified analogue of halichondrin B, has emerged as a widely utilized therapeutic option for patients with doxorubicin-resistant disease (6). Beyond eribulin, the field of liposarcoma therapeutics encompasses a spectrum of drug treatments, including anthracyclines, ifosfamide, gemcitabine, dacarbazine and targeted agents such as trabectedin, tyrosine kinase inhibitors and CDK4/6 inhibitors, reflecting the ongoing efforts to improve patient outcomes in this challenging malignancy (7). In the past decade, immune checkpoint inhibitors (ICIs) have influenced the therapeutic paradigm in various solid cancers. However, their efficacy in liposarcoma remains controversial (8). Dedifferentiated liposarcoma (DDLPS) exhibits a relatively higher mutational burden, T cell infiltration and PD-L1 expression, thus becoming the predominant liposarcoma subtype evaluated in clinical trials (9, 10). In contrast, myxoid liposarcoma (MLPS) shows limited responsiveness to ICIs due to its relatively poorer immunogenicity (11). In a phase II randomized multicenter clinical trial, the objective response rate (ORR) for DDLPS patients treated with nivolumab or nivolumab plus ipilimumab was 6.7% and 14.3%, respectively (12). Currently, there is a lack of data regarding ICI treatment specifically for the MPLPS subtype. Here, we reported a patient with advanced MPLPS who received eribulin plus sintilimab, combined with radiotherapy for last-line therapy, achieved a partial response (PR) and 12 months of progression-free survival (PFS).

## Case description

**Figure 1:**
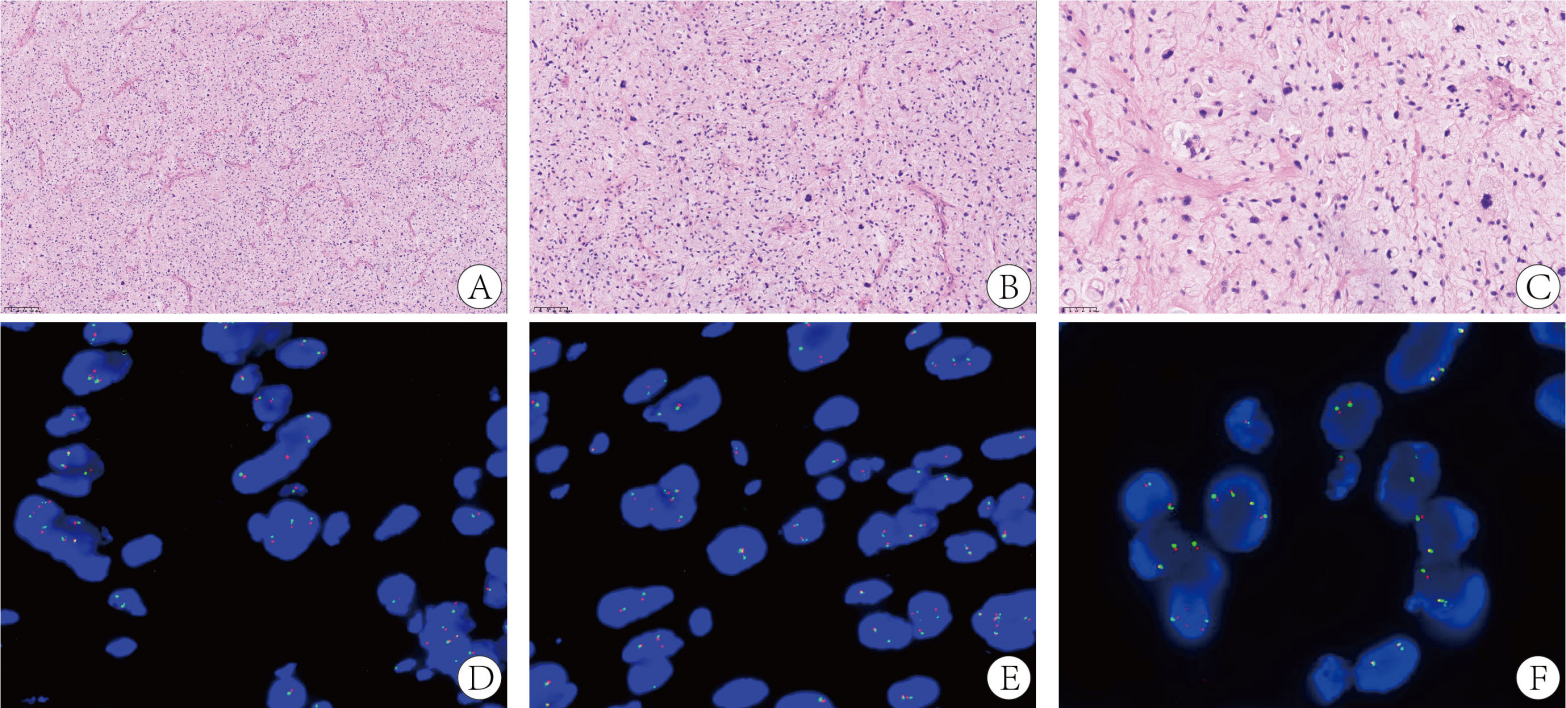
**(A)** The tumor stroma is rich in mucous and branched capillaries, similar to myxoid liposarcoma (H&E staining, ×100). **(B)** The tumor cells have obvious atypia and deep staining nuclei (H&E staining, ×200). **(C)** The tumor cells are pleomorphic and there are some pleomorphic lipoblast cells, similar to pleomorphic liposarcoma (H&E staining, ×400). FISH assay shows no translocation of CDK4 gene **(D)**, FRS2 gene **(E)** and MDM2 gene **(F)**.

**Figure 2:**
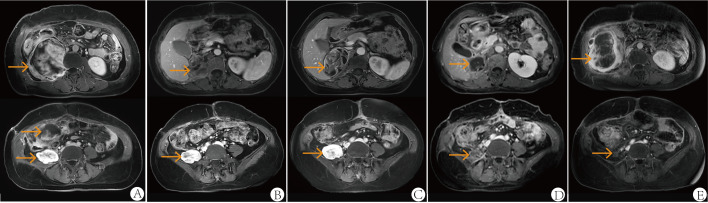
Abdominal MRI at first relapse in October 2021 **(A)**, one month after the second surgery in April 2022 **(B)**, after two cycles of second-line therapy (gemcitabine plus albumin paclitaxel) in June 2022 **(C)**, after six cycles of third-line therapy (eribulin plus sintilimab, combined with radiotherapy) in December 2022 **(D)** and at follow-up in June 2023 **(E)**. All images were acquired with T1-weighted sequences.

In January 2020, a 52-year-old female presented with numbness in the right lower limb. Magnetic resonance imaging (MRI) revealed a large cystic solid mass in the upper segment of the right psoas major muscle. The patient underwent R0 resection of the retroperitoneal tumor, which measured approximately 5 × 5 × 6 cm, with negative surgical margins confirmed by pathological examination. No adjacent organs required en bloc resection. The patient’s recovery was uneventful. Pathological examination established the diagnosis of MPLPS (**Figure 1**). Immunohistochemistry (IHC) staining showed CD34 (partial+), STAT6 (-), myogenin (-), MyoD1 (-), WT1 (partial+), CR (-), TRK (-), ROS1 (-), TLE1 (-), desmin (-), S100 (-), CD117 (-) and DOG1 (-). The tumor was classified as grade 3 based on histopathological findings. FISH assay revealed no translocation of MDM2, FRS2 or CDK4 genes. Next-generation sequencing identified an SDHA mutation (exon7, p. L285R, 6.7%), RUNX1 amplification, AXL amplification, CDKN1A amplification, DNMT3A deletion, FLT3 deletion, MYCN deletion, microsatellite stability (MSS) and a tumor mutational burden (TMB) of 1.92 mut/Mb. Unfortunately, PD-L1 expression was not assessed due to the unavailability of antibody 22C3 at that time. The patient didn’t receive any adjuvant therapy and underwent regular follow-up. One year later, she underwent a surveillance MRI, which revealed multiple nodular soft tissue masses of varying sizes in the right iliac fossa. The cross-sectional size of the largest mass was approximately 5.0cm×4.1cm (**Figure 2A**). Chemotherapy was prescribed after recurrence in October 2021, consisting of doxorubicin (75mg/m^2^, day 1, every 3 weeks) plus ifosfamide (1800 mg/m^2^, days 1-5, every 3 weeks). After two cycles, some tumors increased in size while others decreased. Anlotinib was added to the first-line regimen of doxorubicin and ifosfamide in January 2022, but discontinued after only one cycle due to treatment-related adverse events including persistent severe low back pain, anorexia and weight loss. After four cycles of first-line therapy, the size of the abdominal tumor increased, and the response was evaluated as progression disease (PD).

**Figure 3:**
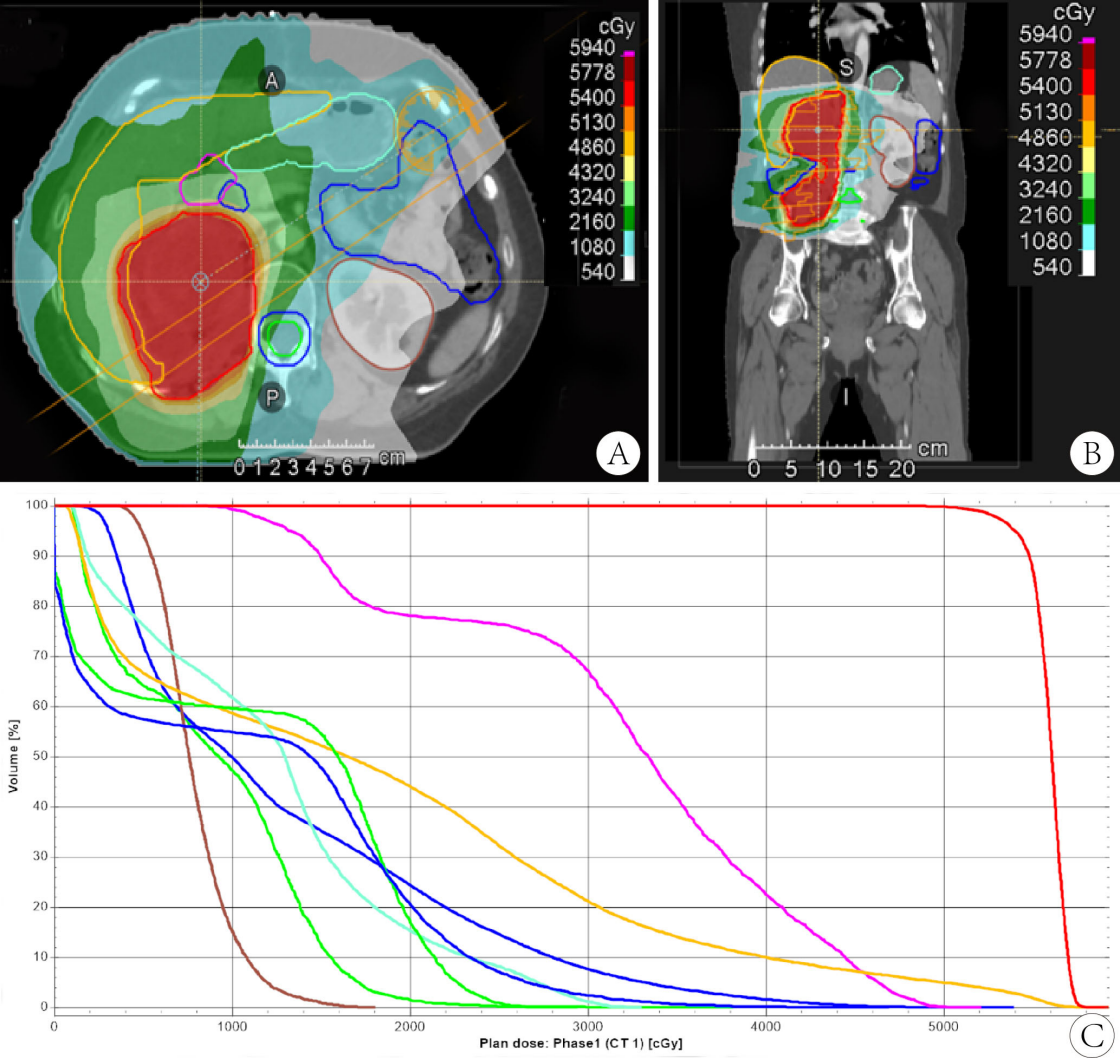
Radiotherapy range was from the 11th thoracic vertebra to the 5th lumbar vertebra **(A, B)** and radiation dose was 54 Gray in total delivered in 18 fractions **(C)**.

**Figure 4:**
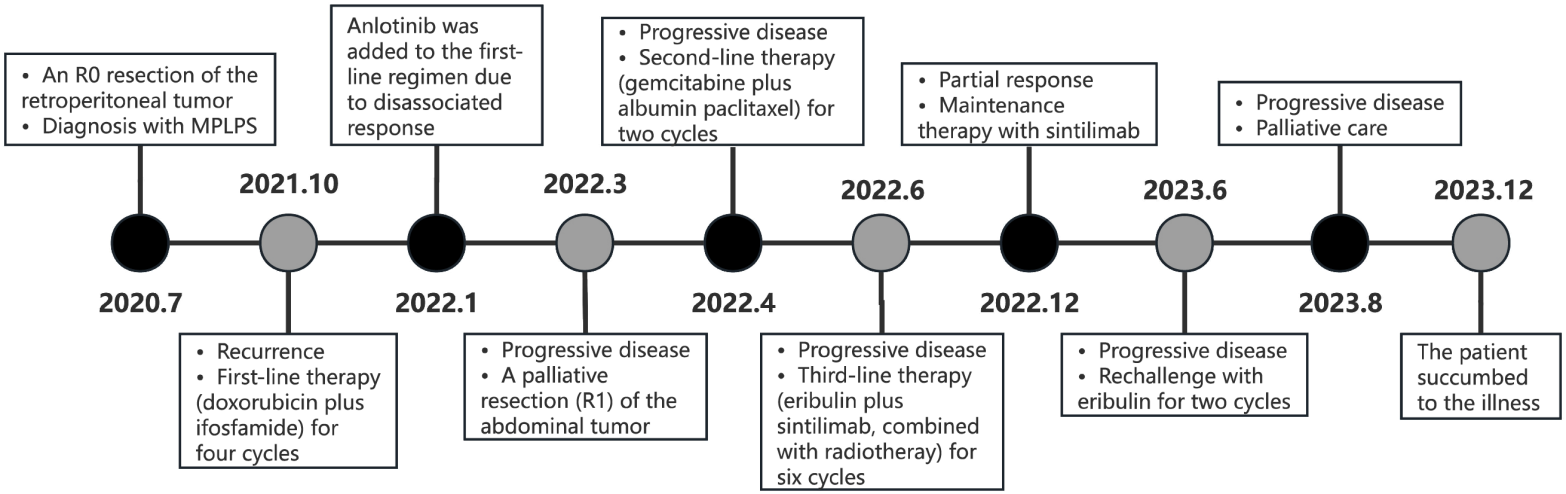
Timeline of the patient treatment process.

Following the patient’s decision to pursue surgical intervention for the abdominal tumor, palliative cytoreductive surgery was performed. Intraoperatively, the tumor was found to be large and densely adherent to surrounding structures, necessitating extensive en bloc resection including the right kidney and right adrenal gland, partial repair of the abdominal aorta and inferior vena cava, limited repair of the colon and adhesiolysis. Final pathological evaluation confirmed an R1 resection margin. However, the MRI showed a rapid increase in tumor volume only one month after surgery (**Figure 2B**). The patient’s recovery from the second procedure was sufficient to permit the initiation of subsequent systemic therapy. In April 2022, the patient started second-line therapy of gemcitabine plus albumin paclitaxel. Unfortunately, the outcome was evaluated as PD after two cycles (**Figure 2C**). After a multi-disciplinary discussion, the patient was started on combination therapy of eribulin (2mg on days 1 and 8, every 3 weeks) and PD-1 inhibitor sintilimab (200mg, every 3 weeks) in June 2022. Concurrently, she underwent low-dose radiotherapy targeting the abdominal tumor. The radiation dose was escalated in coordination with immunotherapy and eribulin. A total dose of 54 Gy was delivered to the gross tumor volume in 18 fractions, covering the region from the upper border of the 11th thoracic vertebra to the lower border of the 5th lumbar vertebra (**Figure 3**). After six cycles of combination therapy, the tumor gradually decreased in size, achieving a partial response (PR) in December 2022 (**Figure 2D**). Treatment-related adverse events included fatigue, abdominal pain, leukopenia, neutropenia, thrombocytopenia as well as elevations in transaminases and uric acid, all graded as CTCAE Grade 1-2. Subsequently, the patient received maintenance therapy with sintilimab from December 2022, which effectively controlled all adverse events. Remission was sustained for 12 months. However, in June 2023, follow-up MRI indicated disease progression, accompanied by the gradual onset of abdominal pain (**Figure 2E**). Despite a rechallenge with eribulin, the disease progressed rapidly, and the patient declined further anti-tumor treatment. Regrettably, the patient succumbed to the disease in December 2023. **Figure 4** illustrates the patient’s treatment course.

## Discussion

According to the WHO classification of soft tissue sarcoma (STS), liposarcoma is divided into five subtypes including atypical lipomatous tumor/well-differentiated liposarcoma(ALT/WDLPS), dedifferentiated liposarcoma(DDLPS), myxoid liposarcoma(MLPS), pleomorphic liposarcoma(PLPS) and MPLPS (1). MPLPS is a newly recognized and extremely rare subtype. Literature on MPLPS is limited, with only 48 case reports documented as of 2024 (13). According to data from the NETSARC+ database, between 2010 and 2023, MPLPS comprised only 0.1% of the 11,132 recorded liposarcoma cases (14). However, no data regarding its incidence in China have been published to date. Morphologically, MPLPS is characterized by a mixture of pulmonary edema-like myxoid cellular and high-grade pleomorphic liposarcoma cellular features (5). Molecularly, MPLPS differs from other subtypes, lacking EWSR1-DDIT3 gene fusions and MDM2 amplifications (15). Clinically, MPLPS is associated with a worse outcome than pleomorphic liposarcoma and myxoid liposarcoma. Dermawan et al. reported a median overall survival (OS) of 22.6 months for MPLPS, compared with 75.9 months for PLPS (*p* = 0.0018) and 218.3 months for MLPS (*p* = 0.0732) (5). Compared with DDLPS, MPLPS demonstrates a more aggressive clinical course and a poorer prognosis, characterized by high rates of both local recurrence and distant metastasis to lung, soft tissue, and unusual sites such as bone (16). Consequently, the treatment sensitivity of MPLPS remains unclear and may differ from that of other liposarcoma subtypes. Generally, MLPS is radio- and chemo-sensitive, whereas PLPS and DDLPS are less responsive to chemotherapy and radiotherapy, and ALT/WDLPS is considered radio- and chemo-insensitive (17, 18). Currently, the treatment regimens for MPLPS are mainly extrapolated from the management of other liposarcoma subtypes.

The first-line treatment for metastatic liposarcoma is based on doxorubicin with or without ifosfamide, like other STS subtypes. In the randomized phase III trial EORTC 62012, the combination of doxorubicin and ifosfamide showed significantly higher progression-free survival (PFS) and objective response rate (ORR), but no benefit in OS compared with doxorubicin alone in patients with advanced or metastatic soft tissue sarcoma (19). Multivariate analysis indicated that liposarcoma was associated with a higher tumor response rate compared with other histological subgroups (20). However, the specific liposarcoma subtypes were not described in this trial. With the purpose of tumor shrinkage and symptom relief, our patient received doxorubicin plus ifosfamide as first-line therapy. However, the tumor progressed after only 4 cycles.

For second-line treatment, chemotherapy regimens such as dacarbazine, gemcitabine-docetaxel and targeted therapies have been widely applied in various STS subtypes including liposarcoma (7). Trabectedin and eribulin are specifically approved by FDA for unresectable or metastatic liposarcoma following progression on anthracycline-containing treatment. A phase III randomized multicenter clinical trial compared trabectedin with dacarbazine in patients with advanced liposarcoma or leiomyosarcoma after failure of anthracycline-based regimens. Trabectedin resulted in a longer mPFS compared with dacarbazine (3.0 vs. 1.5 months) in patients with liposarcoma, with the benefit being most pronounced in the MLPS subgroup (5.6 vs. 1.5 months) (21). Unfortunately, trabectedin has not been marketed in China.

Eribulin, an inhibitor of microtubule polymerization, was approved for anthracycline-pretreated patients with metastatic liposarcoma by the FDA and EMA and for all STS subtypes in Japan (6). In a randomized phase III study (NCT01327885), eribulin demonstrated a longer median OS than dacarbazine (13.5 vs 11.5 months, *p* = 0.0169) in previously treated patients with advanced liposarcoma or leiomyosarcoma (22). Subgroup analysis revealed that eribulin significantly improved median PFS (2.9 vs. 1.7 months, p = 0.0015) and median OS (15.6 vs. 8.4 months, p < 0.001) compared with dacarbazine in previously treated liposarcoma patients (23). Notably, eribulin demonstrated significantly longer OS in patients with PLPS (22.2 vs. 6.7 months) and DDLPS (18.0 vs. 8.1 months) compared with dacarbazine, while no significant difference in OS was observed in patients with MLPS (13.5 vs. 9.6 months). These findings suggest a varied treatment response to eribulin among different subtypes of liposarcoma.

Pazopanib, a multitarget tyrosine kinase inhibitor (TKI), was evaluated in a single-arm phase II trial (EORTC 62043) in patients with relapsed or refractory advanced STS. However, due to the histological misclassification in the first stage, the progression-free rate(PFR) at 12 weeks in the adipocytic sarcoma cohort was only 17.6%, leading to the exclusion of liposarcoma from the second stage of this trial and the subsequent phase III trial (PALETTE) (24, 25). A *post hoc* analysis showed that after a centralized histopathological review, two patients were re-assigned to the liposarcoma cohort and the corrected PFR at 12 weeks met the pre-defined threshold of >20%(26%), suggesting that liposarcoma should not have been excluded from further evaluation. In a subsequent phase II study including 52 liposarcoma patients, pazopanib yielded a mPFS of 3.5 months for WDLPS/DDLPS and 1.9 months for MLPS, resulting in early termination of the MLPS cohort due to insufficient efficacy. Another single-arm phase II trial investigated pazopanib in 41 patients with advanced liposarcoma, showing different results with 12-week PFR of 74.1% for DDLPS and 66.7% for MLPS (26). Anlotinib, a novel multitarget TKI which inhibits VEGF/VEGFR, PDGFRa/b, c-Kit, Ret, Aurora-B, c-FMS and DDR1 (27), showed antitumor activity in advanced liposarcoma in a multicenter phase II trial. The median PFS and OS for liposarcoma were 5.6 and 13 months, respectively. Based on these results and the results of subsequent phase IIb trial (ALTER0203), anlotinib has been approved in China for advanced STS including liposarcoma after anthracycline pretreatment (28, 29). Our previous retrospective study evaluated the efficacy and safety of anlotinib in patients with unresectable or metastatic well-differentiated/dedifferentiated liposarcoma. The estimated median PFS and OS were 27.9 and 56.6 weeks respectively, with a disease control rate of 64.7% (30). Other multitarget TKIs such as sunitinib, sorafenib and regorafenib have shown limited efficacy for advanced liposarcoma (31–33). Unfortunately, our patient couldn’t tolerate the adverse effects of anlotinib.

ICIs have revolutionized the treatment landscape for various solid cancers including some subtypes of sarcoma. The immunological environment of LPS is highly heterogeneous across different subtypes. DDLPS is characterized by a higher abundance of infiltrating T cells and PD-1 expression, yet MLPS has a relatively cold immune microenvironment. The multicenter, single-arm phase II trial SARC028 evaluated the safety and efficacy of the anti-PD-1 antibody pembrolizumab for advanced sarcoma. Results indicated that pembrolizumab had efficacy for undifferentiated pleomorphic sarcoma (UPS) and DDLPS (8). However, expansion cohort data for DDLPS were disappointing, with a median PFS of 2 months, median OS of 13 months and ORR of 10% (4/39) (34). Unfortunately, no clinical trial data exist for ICIs in MLPS, PLPS or MPLPS. Overall, alternative strategies are needed to enhance the immune response in liposarcoma. Several studies have explored combining ICIs with systemic chemotherapy, targeted agents or radiotherapy to convert tumor microenvironment from immune-cold to immune-hot. For example, cytotoxic chemotherapy could modulate the tumor immune microenvironment by shifting tumor-associated macrophages (TAMs) toward an anti-tumor M1 phenotype, promoting neoantigen production and enhancing anti-tumor immune responses. Preclinical studies suggest that eribulin may improve the immunotherapy efficacy through modulation of STING signaling. In a phase II study (NCT03899805) evaluating the combination of pembrolizumab and eribulin in patients with STS, only the LPS cohort (n=20) met the primary endpoint of 12-week PFS rate >60% (69.6%), including 17 patients with dedifferentiated liposarcoma (2 PR, 11 SD, 4 PD), 2 patients with pleomorphic liposarcoma (1 PR, 1 PD) and 1 patient with myxoid liposarcoma (SD). The mean PFS for LPS was 31.7 weeks. This combination showed promising activity in the treatment of liposarcomas.

Radiotherapy has been suggested to promote the release of tumor neoantigens and proinflammatory cytokines, potentially synergizing with ICIs (35–38). One retrospective study analyzed the expression of 35 immune response-related genes from 38 sarcoma patients before and after radiotherapy. It showed an upregulated expression of several immune effectors and cancer-testis antigens after radiotherapy, along with a downregulation of immune suppressors (36). Snow et al. characterized the tumor microenvironment in liposarcoma before and after radiotherapy. It has been reported that 63.6% of the patients with DDLPS had increased tumor-infiltrating lymphocyte (TIL) scores and 81.8% had increased inflammation grades after radiotherapy (38). Several ongoing clinical trials are evaluating the efficacy of radiotherapy plus ICIs in STS, primarily focusing on the perioperative management, with few trials focus on advanced STS. A randomized phase II study (NCT03307616) compared the efficacy of neoadjuvant nivolumab or nivolumab plus ipilimumab combined with radiotherapy in patients with UPS or DDLPS (39). The pathological response (percent hyalinization) was 8.8% in DDLPS and 89% in UPS. After neoadjuvant radiotherapy and immunotherapy, tumor-infiltrating B cells increased and were correlated with overall survival in DDLPS patients. Before treatment, B cell infiltration in tumor was associated with higher regulatory T cell density, a relationship that disappeared after ICI therapy. Therefore, the potential of radiotherapy to potentiate antitumor responses represents a rationale for exploring its combination with immunotherapy in liposarcoma, although this synergistic effect specifically in MPLPS requires further validation (40). However, there is currently no clinical trial focusing on MPLPS. Available case reports on MPLPS lack descriptions of systemic therapy (2–4). The optimal treatment strategy for MPLPS remains unknown. Given its poorer outcome, MPLPS appears to require a novel treatment approach distinct from other subtypes of liposarcoma. Our case suggests that the combination of eribulin and PD-1 inhibitor, along with radiotherapy, may be a promising treatment option for advanced MPLPS.

## Conclusion

MPLPS remains an extremely rare neoplasm. Our case presents the most detailed clinical characteristic, imaging features, pathological and treatment data reported to date. This combination regimen demonstrated limited toxicity, and our case exhibited a partial response maintained for 12 months. Overall, this case contributes to the expanding body of knowledge concerning MPLPS and underscores the significance of a multidisciplinary approach in tailoring treatment strategies for rare and challenging soft tissue sarcomas. However, given that this is a single case report, the generalizability of these findings is inherently limited. The observed outcomes were likely influenced by this specific patient’s tumor biology and individual factors, which may not be generalizable. Further research and clinical trials are warranted to elucidate the efficacy and safety profile of this emerging treatment approach for MPLPS patients.

## Data Availability

The original contributions presented in the study are included in the article. Further inquiries can be directed to the corresponding author.
